# In Vivo Assessment of Healing Potential of Ointments Containing Bee Products, Vegetal Extracts, and Polymers on Skin Lesions

**DOI:** 10.3390/ph18010065

**Published:** 2025-01-09

**Authors:** Calin Vasile Andritoiu, Cristina Lungu, Camelia Elena Iurciuc (Tincu), Corina Elena Andriescu, Corneliu Havarneanu, Marcel Popa, Magdalena Cuciureanu, Liliana Mititelu Tarţău, Bianca Ivanescu

**Affiliations:** 1 Apitherapy Medical Center, Balaneşti, 217036 Gorj, Romania; dr_calin_andritoiu@yahoo.com; 2 Nutrition and Dietetics Specialization, Faculty of Pharmacy, Vasile Goldis Western University of Arad, 310025 Arad, Romania; 3 Department of Pharmaceutical Botany, Faculty of Pharmacy, “Grigore T. Popa” University of Medicine and Pharmacy, 700115 Iaşi, Romania; biancaivanescu@yahoo.com; 4 Department of Natural and Synthetic Polymers, Cristofor Simionescu Faculty of Chemical Engineering and Environmental Protection, Gheorghe Asachi Technical University of Iaşi, 700050 Iaşi, Romania; marpopa2001@yahoo.fr; 5 Department of Pharmaceutical Technology, Faculty of Pharmacy, “Grigore T. Popa” University of Medicine and Pharmacy, 700115 Iaşi, Romania; 6 Department of Pathology, Sf. Spiridon Emergency County Hospital, 700111 Iaşi, Romania; andriescu_corina@yahoo.co.uk; 7 Faculty of Psychology and Education Sciences, Alexandru Ioan Cuza University, 700554 Iaşi, Romania; hcornel@uaic.ro; 8 Academy of Romanian Scientists, 050094 Bucharest, Romania; 9 Department of Pharmacology, Grigore T. Popa University of Medicine and Pharmacy, 700115 Iaşi, Romania; mag.cuciureanu@umfiasi.ro (M.C.); liliana.tartau@umfiasi.ro (L.M.T.)

**Keywords:** wound healing, apitherapy products, natural polymers, vegetal extracts

## Abstract

**Background/Objectives:** The present experiment aimed to formulate four ointments that included mixtures of plant extracts (*Hippophae rhamnoides*, *Calendula officinalis*, *Arctium lappa*, and *Achillea millefolium*), apitherapy products (honey, propolis, and apilarnil) and natural polymers (collagen, chitosan, and the lyophilisate of egg white) in an ointment base. **Methods**: In order to investigate the therapeutic properties of the ointments, experimental in vivo injury models (linear incision, circular excision, and thermal burns) were performed on laboratory animals, namely Wistar rats. The treatment was applied topically, once a day, for 21 days. Clinical and macroscopic evaluation, determination of lesion shrinkage rate, re-epithelialization period, and histopathological examination were performed. **Results**: The results demonstrate that the tested ointments have a significant effect in healing skin lesions. On the ninth day of treatment, the wound contraction rate was 98.17 ± 0.15% for the mixed ointment group, compared to the negative control group’s rate of 14.85 ± 2.95%. At day 21, dermal collagenization and restoration of histological structure occurred for all treated groups. **Conclusions**: The tested ointments exerted in vivo wound healing and re-epithelialization effects on incision, excision, and thermal burn injuries.

## 1. Introduction

Wound healing is a process involving distinct overlapping phases of tissue homeostasis, inflammation, proliferation, and remodeling [1]. The healing processes start from the moment of the injury and end with the formation of the scar. The healing process consists of three main stages. First, inflammatory cells are found at the site of the injury. Later, fibroblasts appear that start to produce connective collagen fibers that give tensile strength to the regeneration tissue. Simultaneously, numerous capillaries begin to form to supply nutrients and oxygen to the lesion, while epithelial cells at the edge of the lesion begin to deposit in the area beneath the crust. In the third and last phase, the new epithelium is formed and the wound is considered healed [2]. If any of this stage is not sufficiently completed, wound healing can be impaired, resulting in a chronic injury. Bacterial infection can also delay wound healing [3].

One of the crucial stages in wound healing is the formation of granulation tissues that are able to fill the tissue gap in the proliferation phase, promoting skin regeneration. Chitosan has been shown to promote the proper formation of granulation tissue, accompanied by angiogenesis and the regular deposition of thin collagen fibers. Granulation tissue is composed of new blood vessels, collagen, and several types of cells, including fibroblasts, myofibroblasts, and macrophages [4].

Angiogenesis is the process of forming new blood vessels, which leads to a temporary increase in the number of blood vessels at a site of injury. This process is a crucial part of the proliferative stage of wound healing. Certainly, new capillaries are necessary to bring nutrients, immune cells, and oxygen to healing wounds, and defects in angiogenesis are frequently associated with delays in wound healing [5].

Angiogenesis provides nutrients to the healing tissue and induces structural repair through the formation of granulation tissue. For example, sea buckthorn seed oil stimulates angiogenesis, a fact highlighted by the increased expression of VEGF (vascular endothelial growth factor)*,* as reported in the specialized literature [6].

The use of natural molecules and polymers as remedies or for tissue engineering is, in fact, a major approach for tissue regeneration [7]. Recently, interesting stimulation effects on the physiology of human skin cells by using plant polysaccharides have been demonstrated [8,9].

There is growing evidence that established therapies’ impacts depend on their capacity to release a mix of factors that promote tissue regeneration, modulate the local environment, and encourage the proliferation, migration, differentiation, survival, and functional recovery of resident cells [10,11].

The search for natural compounds that can stimulate tissue regeneration has gained importance in recent years, aiming at the development of non-toxic formulas for treating wounds. In this regard, the inclusion of apitherapeutics, plant extracts, and natural polymers is beneficial. Results obtained in previous studies that aimed to evaluate in vivo the wound healing potential of ointments with plant extracts, apitherapeutic products, and natural polymers encouraged the present study [12,13,14]. Thus, ointments based on bee products (propolis, honey, apilarnil) showed positive effects on the healing period, but also on the histological architecture of the skin tissue [14]. At the same time, four ointments based on vegetal extracts (*Achillea millefolium*, *Arctium lappa*, *Calendula officinalis*, and *Hippophae rhamnoides*) were tested on the same wound models, obtaining dermal collagenization at day 21 of treatment [12]. The ointments based on polymers (chitosan, collagen, and lyophilized egg white) demonstrated good hemostasis on the skin tissue and good efficiency on the newly formed epithelization tissue [13].

In this context, the purpose of the present study is to evaluate the synergistic effects of four ointments based on the same apitherapeutic products, vegetal extracts, and natural polymers (but mixing them together in different formulations) on wound healing, using similar experimental animal models (linear incision, circular excision, and thermal burn).

## 2. Results

### 2.1. Wound Healing Evaluation Parameters

#### 2.1.1. Macroscopic Evaluation

The tested ointments containing bee products, plant extracts, and natural polymers have proven their effectiveness. Clinical, macroscopic healing occurred after six days of treatment, in the case of the incision model, and after nine days in the case of the excision models and in the thermal burn injury (the images are included in the Supplementary Information file (Table S1)).

#### 2.1.2. Period of Re-Epithelialization and Wound Contraction Rate

The wound areas were measured on days 0, 6, and 9. The results are presented in Table 1.

There were no significant changes in wound contraction during the first three days of treatment (these are the days when the inflammatory processes occur). Cell proliferation began after 3 days, and a significant reduction in wound area was obtained at 6 and 9 days. It was observed that the treatment with the APPo ointment showed the best effects, as it recorded the best rate of contraction of the lesions on the sixth day of the experiment (91.35 ± 0.83%) compared to the OB group (26.74 ± 2.13%) and especially compared to the NC group (5.78 ± 1.86%). On the ninth day of treatment, the results were obviously positive; for the APPo group, the percentage of wound contraction was 98.17 ± 0.15% compared to 33.79 ± 2.21% (OB group) or 14.85 ± 2.95% for the NC group. On the 12th day of treatment, the re-epithelialization process was complete for the wound excision model for the groups treated with the four ointments.

The synergistic effects regarding the healing of the excision-type lesion can be observed in the present study for the APPo group, which, on the ninth day of treatment, showcased a lesion area of only 1 × 1 mm, followed by the APo group (1 × 1.5 mm). It can be seen that the groups treated with ointments in which bee products were included (AP, APo, APPo) occupy the first three places in the hierarchy of healing.

#### 2.1.3. Histopathological Examination

The relevant pathological anatomy results are presented for each group on days 1, 2, 3, 9, and 21 of the study (Table 2, Table 3, Table 4, Table 5 and Table 6).

On the first day of the experiment, when the experimental models were made, in excision and thermal burn, the following were noted: 1–2 lymphocytes, respectively, 2–3 fibroblasts, 1 capillary vessel/high-power field—HPF; discrete edema and discrete fibrosis (accentuation of basement membranes with horizontalization and homogenization of some collagen bundles) in incision and excision. In the thermal burn, severe fibrosis of the dermis and burnt epidermis could be distinguished. In the incision-type lesion, an intracornous abscess with spongiosis and dehiscence of the epidermis could be seen at the epidermis level (Table 2).

On the second day of treatment, similar aspects were found in incision, excision and thermal burn: 2–3 lymphocytes/HPF, 3–4 fibroblasts/HPF, 1 vessel/HPF. In the incision-type lesion, for the groups treated with test ointments, the presence of an inflammatory infiltrate of various degrees in the hypodermis, which includes the muscle tissue, was noted. On the other hand, for the NC and OB groups, the histological images were much more pronounced, with the presence of an important inflammatory infiltrate at the hypodermis level, with the interest of the striated muscle. For the excision lesion, the following were found: inflammatory infiltrate in the hypodermis (PPo group), vessels with thickened walls (AP group), intracorneal abscess (APo group), ulceration of the lesion (NC group), and aspects of myositis (OB group). In the thermal burn, aspects of intense dermal collagenization were highlighted for all the groups studied, with hemorrhage (OB group) and inflammatory infiltrate in the hypodermis that also affected the muscle layer (Table 3).

For the third day of treatment, for the groups treated with ointments, in the incision-type lesion, an inflammatory infiltrate in the dermis and hypodermis (AP, PPo, APo groups) was noted. In addition, microulceration of the epidermis was observed for the APPo group, which also showed hematoleukocyte surface deposit. For the excision-type lesion, the presence of inflammatory lymphocytic infiltrate (AP, PPo, APPo groups) was observed, which also affects the muscle layer (PPo group). For the APo group, a corneous abscess with vacuolization was present. In the burn-type lesion, fibrosis embedding the striated muscle fibers (AP), abscess in the stratum corneum (PPo), congestion and hemorrhage (APo), and inflammatory infiltrate (APPo) could also be identified (Table 4).

For the control groups, on the third day, for the incision-type lesion, stratum corneum abscess, epidermal acanthosis (NC group), and keratinocyte vacuolar degeneration (OB group) were observed. For the excision-type lesion, the presence of ulcers (NC group) and hypodermic inflammatory infiltrate (OB group) were noted, and for the burn-type lesion, the presence of abscess in the stratum corneum was noted for both groups (NC and OB) (Table 4).

On the ninth day, for the incision-type injury, all groups treated with ointments (AP, PPo, APo, APPo) showed dermal collagenization. For the excision-type lesion, dermal collagenization was also noted, except for the AP group, which presented severe dermal collagenization that also affected the muscle plane. In the burn-type lesion, dermal collagenization was observed for all treated groups and a moderate inflammatory infiltrate was present for the APo and APPo groups (Table 5).

For the NC group, an important inflammatory infiltrate was identified in the incision and in the excision edema that also affected the muscle plane. For the OB group in incision and excision, a giant cell foreign body reaction to keratin was evident, and in the thermal injury, an inflammatory infiltrate at the level of the layers of skin tissue also affected the muscle plane (Table 5).

At day 21, for groups that were treated with the tested ointments, dermal collagenization for the PPo and APPo groups was observed in all three types of skin lesions, which was also noticed for APo group in incision and excision. For the AP group, significant dermal collagenization was found for incision and thermal burn. For excision, in addition to this collagenization, the presence of discrete dermal edema was also observed. For the APo group, dermal collagenization was observed in the excision but with highlighting of the hair follicle and atrophy of the sebaceous glands (Table 6).

For the other groups, an inflammatory infiltrate was evident in the incision, which affected the hypodermis and the muscular layer (NC group). For OB group, the presence of fibrosis in the hypodermis was also noted. In addition to this, foreign body-type giant cell was also evident. In excision, an important inflammatory infiltrate was noted for both groups, and, for NC, the presence of ulceration aspects was also noted. For the burn-type injury, there was an evident inflammatory infiltrate that also affected the muscle plane (NC group), congestion, and edema (OB group) (Table 6).

## 3. Discussion

The newly tested ointments containing bee products, plant extracts and natural polymers were effective in healing all three types of wound models. Clinical, macroscopic healing occurred after six days of treatment, in the case of the incision model, and after nine days in the case of the excision and thermal burn injury models. Right from the first day of treatment with the ointments containing apitherapeutic products, plant extracts, and natural polymers, the effect on hemostasis was observed.

The re-epithelialization area was 1 mm^2^ on day 9 of treatment, compared to other previously tested groups where the re-epithelialization period was observed and measured on day 12. Also, the WCR values (91.35 ± 0.83 on day 6 and 98.17 ± 0.15 on day 9) were the highest compared to previous studies [12,13,14].

We found superior results on days 6 and 9, both in terms of wound contraction and re-epithelialization, with the best results being obtained with the ointment that assumed the synergistic effect of apitherapy–phytotherapy–natural polymers principles. These effects are supported, in addition, by the histological results, where, from the ninth day, for the treated groups, dermal collagenization is observed, sometimes important (AP group) or dermal collagenization with rare inflammatory elements. On day 21, dermal collagenization and restoration of histological structure occurred for all treated groups, and healing occurred without keloid scars.

The mechanism of hemostasis mediated by collagen is the subject of scientific investigations in the healthcare sector. Collagen binds to platelet receptors and activates further adhesion of platelets, forming an aggregate. Collagen is one of the main catalysts of the platelet response after injury; it promotes thrombocyte production and adhesion, intervenes in the complete activation of platelets, and also initiates the coagulation pathway [15].

It is known that chitosan has hemostatic properties, by inducing thrombocyte adhesion and promoting erythrocyte and platelet aggregation. Chitosan also inhibits the dissolution of fibrin, hence prolonging hemostasis [4]. Activation of the coagulation cascade is an important factor in acute, deep injuries, when excessive bleeding can be life-threatening. These properties have led to the use of chitosan as a compound in functional wound dressings to improve the wound healing process or, more precisely, to accelerate wound healing [16,17,18].

As indicated by the study results, from the second day of treatment, the tested ointments, based on natural products, showed effects on fibroblasts. The specialized literature reports that chitosan stimulates the migration and proliferation of fibroblasts, increases angiogenesis, and stimulates the synthesis of glycosaminoglycans, proteoglycans, and collagen [4].

After three days of treatment, the test ointments demonstrated significant anti-inflammatory and anti-infectious effects in all three types of lesions. By stimulating inflammatory cells, macrophages, and fibroblasts, chitosan enhances the inflammatory phase, which therefore ends sooner, allowing the regeneration phase to start more quickly [17]. Chitosan and its derivatives are intensively used in regenerative medicine due to their superior antibacterial properties. The antimicrobial properties of chitosan are thought to be due to the interaction between NH_2_ groups that are positively charged and negatively charged components on bacterial cell surfaces. This interaction leads to the disruption of the microbial membrane/cell wall and subsequently to the leakage of intracellular constituents [4,17].

The inclusion of antibacterial agents is a strategy used to improve the wound healing process, and for the present study, we also included propolis, honey, and vegetal extracts. Regarding the infectious process, it is known that the exposure of an injury to the external environment worsens the healing phases because the wound is exposed to microbial attack. The two most common bacteria found in chronic wounds are *Pseudomonas aeruginosa* (Gram-negative) and *Staphylococcus aureus* (Gram-positive). These bacteria can accumulate on the wound surface and penetrate the underlying tissues. Wound infection can significantly compromise the healing process and, in some cases, stop it [17].

Propolis demonstrates a broad array of biological effects such as antimicrobial, local anesthetic, antioxidant, immunostimulatory, and anti-inflammatory. It has been used for wound healing, tissue regeneration, treatment of burns, mouth ulcers, trophic ulcers, and surgical ulcers [19,20]. In addition to its tissue regeneration property, propolis has proven effects against numerous pathogenic microorganisms [21]. Patients with infected lesions who benefited from local treatment with propolis and a systemic antibiotic showed better progress than those treated only with an antibiotic [19]. Furthermore, various formulations with propolis used in dressings after surgery lead to faster healing of scars and wounds [20].

Additionally, it has been demonstrated that the topical application of honey leads to the rapid eradication of bacterial infections, reduces the use of antibiotics and the period of hospitalization, and accelerates the healing of wounds, with the result of minimal scaring [22]. Different factors contribute to the antibacterial efficacy of honey: its high sugar content, acidity, and low water activity; the polyphenol compounds; hydrogen peroxide; and bee peptide defensin-1 [23].

Moreover, the use of plants in wound healing has been described in various traditional medicine systems, such as Ayurveda and traditional Chinese medicine. In addition to single-plant extracts or isolated compounds, multi-plant formulations have been tested to evaluate wound healing efficacy. It has been noted that the wound healing potential of plants often correlates with the potential to induce angiogenesis, which is an essential step in wound healing. Recently, most of these plants have been studied in order to establish the impact on proangiogenesis in vitro and in vivo, with particular attention being paid to the expression of VEGF and its receptor, VEGFR2. Numerous plants and herbal products have wound healing properties [24]; however, for the present study, we used an original formula, and, in addition, we incorporated both the hydroalcoholic and the oily extract into the ointment.

Of note for the present study is the collagenization effect that the test ointments, containing natural compounds, exhibited in all three types of lesions. It is known that bee products contain a quantity of arginine, which is considered to have a major influence on collagen and the improvement of the immune reaction, thus contributing to healing [25,26,27]. Considering this collagenization at the level of the lesions, we can intuit that the test ointments helped maintain a good level of hydroxyproline, understanding that a deficiency in hydroxyproline impairs collagen synthesis and fibroblast production [28].

It can be noted that the test ointments contributed to the normalization of the histoarchitecture of the skin tissue, which confirms the already published studies. Studies have shown that chitosan stimulates cell proliferation and tissue regeneration [29] and, due to its ability to improve the remodeling phase of the extracellular matrix, it can be incorporated into formulations for treating wounds [30]. A study suggests that chitosan and its nanoparticles facilitate the extracellular matrix in the remodeling phase of wound healing [31]. The main factor in accelerating the wound healing process can be attributed to the presence of N-acetyl-d-glucosamine [32]. Chitosan also promotes growth factors or cytokines in an early phase of healing, thus contributing to angiogenesis [30].

Another compound that has led to the fast and safe healing of skin tissue is CAPE (phenethyl ester of caffeic acid), an active component of propolis. CAPE accelerates wound healing and re-epithelialization by decreasing oxidative stress [33]. CAPE can enhance wound healing through its antioxidant and anti-inflammatory properties [34].

We have reported in previous papers the results of HPLC analysis of the propolis extracts, and the hydroalcoholic extracts of *Arctium lappa*, *Achillea millefolium*, and *Calendula officinalis*, which we also used in the formulation of the tested ointments [12,14]. The main phenolic compounds in the hydroalcoholic propolis extract were ferulic acid and p-coumaric acid (1771.669 µg/mL and 1516.119 µg/mL, respectively). Moderate amounts of flavones (apigenin and luteolin), flavonols (quercitin and kaempferol), and quercitin glycosides (quercitrin, isoquercitrin, and rutoside) were also reported. In the oily extract of propolis, we identified the presence of three phenolic acids (ferulic acid, p-coumaric acid, and caffeic acid) [14].

HPLC analysis of the hydroalcoholic extracts of *Bardanae folium*, *Millefollii herba*, and *Calendulae flos* revealed the presence of caffeic acid, chlorogenic acid, and rutoside in all three extracts. Moreover, *Achillea millefolium* extract also contains small quantities of quercitrin, quercetin, luteolin, and apigenin. Isoquercitrin was reported in *Calendula officinalis* extract, and ferulic acid in *Arctium lappa* extract [12].

Ferulic acid and p-coumaric acid, the main compounds identified in propolis extract, promote wound healing through complementary molecular mechanisms involving antioxidant, anti-inflammatory, antimicrobial, and regenerative properties. Ferulic acid reduces inflammation by inhibiting the NF-κB pathway and downregulating pro-inflammatory cytokines like TNF-α and IL-6 while also suppressing COX-2 and iNOS expression. It scavenges reactive oxygen species (ROS), upregulates antioxidant enzymes like glutathione peroxidase (GPx), and protects fibroblasts and keratinocytes from oxidative damage. P-Coumaric acid also counters reactive oxygen species (ROS) and boosts antioxidant enzymes like superoxide dismutase (SOD) and catalase (CAT), playing a role in cellular defense. Both compounds stimulate fibroblast proliferation, keratinocyte migration, and collagen synthesis, but ferulic acid uniquely inhibits matrix metalloproteinases (MMPs), preventing excessive ECM degradation. Additionally, ferulic acid upregulates TGF-β1 and VEGF to accelerate angiogenesis, while p-coumaric acid primarily enhances VEGF expression to improve oxygen and nutrient delivery. Their antimicrobial activities prevent infections, creating an optimal environment for wound repair and tissue regeneration [35,36].

In addition, other phenolic acids and the flavonoids found in the propolis and plant extracts promote wound healing through similar activities. They scavenge reactive oxygen species (ROS) and enhance endogenous antioxidant enzyme activity, protecting cells from oxidative stress. By inhibiting the NF-κB pathway, flavonoids reduce the production of pro-inflammatory cytokines like TNF-α and IL-6, alleviating inflammation. They stimulate fibroblast proliferation, keratinocyte migration, and collagen synthesis, which are essential for extracellular matrix remodeling and tissue regeneration. Flavonoids also promote angiogenesis and improve blood supply to the wound. Moreover, their antimicrobial effects prevent wound infections [37].

Another bee product that contributed synergistically to the results obtained in healing injuries was honey. In addition to its content of phytohormones and bioactive factors, the value of honey in therapeutics lies primarily in its emollient qualities and in its ability to determine by osmosis an increased influx of blood to the skin tissues, thus improving their nutrition. At the skin level, glucose freely diffuses into the interstitial fluid of the dermis and epidermis, from where it reaches the cells. Over half of the glucose used in the skin occurs in the epidermis [38].

Apart from substances with a role in obtaining energy at the cellular level, carbohydrates form, together with other groups of substances, complex molecules with a structural role in the architecture of the cell. Thus, in the epidermis, as in other tissues, glycoproteins, glycolipids, glycoaminoglycans, proteoglycans, and nucleic acids are synthesized [38]. Honey stimulates the growth of wound tissue, accelerates healing, and produces debridement of the lesional crust [39]. In our study, it was observed that the groups treated with ointments in which bee products were included occupied the first three places in the hierarchy of healing [Table 1]. We can conclude that they play an important role in wound healing.

In general, an effective wound dressing maintains a moist wound bed environment without risking dehydration or exudate accumulation; it has sufficient permeability for gases and reparative processes that require oxygen, and a high level of fluid absorption capacity to remove excessive exudates containing nutrients for bacteria from the wound; it is a good barrier against the penetration of microorganisms that can cause infections; it has antibacterial activity to suppress the growth of bacteria under the dressing; it lacks cytotoxic effects. The ointments tested in the present study successfully proved they are effective wound dressings.

In terms of limitations, a problem could be the source of bee products and medicinal plants. The concentration of active principles in plants may vary depending on pedo-climatic conditions and our extracts were not standardized. In this study, we used propolis, honey, and apilarnil all collected from the same apiary (S.C. Stupina SRL, Balanesti, Gorj County, Romania). However, it is difficult to ensure reproducibility and standardization if sources from different areas are used. Another aspect that must be taken into account is sensitization reactions in patients with allergies to plants from the *Asteraceae* family and to bee products. Therefore, the ointments must undergo sensitization and irritation testing in order to ensure their safety.

In future research, we aim to resolve the limiting aspects mentioned above and to expand the investigations by evaluating the therapeutic potential of some biofilms with controlled release of active principles from plant extracts, bee products, and natural polymers at the level of skin tissue, including on a diabetic wound model as well.

## 4. Materials and Methods

### 4.1. Plant Material

The flowers of *Achillea millefolium* L. and *Calendula officinalis* L., the leaves of *Arctium lappa* L., and the fruits of *Hippophae rhamnoides* L. were collected from Balanesti (Gorj, Romania). The plant material was identified and authenticated by specialists from the Department of Pharmaceutical Botany, “Grigore T. Popa” University of Medicine and Pharmacy, Iasi, Romania. Voucher samples (CO2016, AM2016, AL2016, and HR2016) are deposited in the Department of Pharmaceutical Botany.

### 4.2. Preparation of the Oily and Hydro-Alcoholic Extracts

To obtain the oily extracts, we macerated 50 g of fresh and ground plant material (*Bardanae folium*, *Millefolli herba*, and *Calendulae flos*) with 500 mL of virgin olive oil (Monini S.p.A. SS, Spoleto, Italy) for two weeks at room temperature, while the oily extract of *Hippophae fructus* was obtained from a commercial source (SC Hofigal Export Import SA, Bucharest, Romania). Later, the four extracts were mixed in equal proportions, obtaining an extract named OVE. The four hydro-alcoholic extracts were also obtained using the maceration method, in which 50 g of dried and ground plant were kept in contact with 500 mL ethanol 70% *v*/*v* for two weeks at room temperature. Then, they were pooled in equal amounts, obtaining an extract named HVE [12].

### 4.3. Preparation of the Ointments

#### 4.3.1. Preparation of the Ointment with Vegetable Extracts and Bee Products

In 100 g of ointment base (50 g of vaseline and 50 g of lanolin), 15 mL of OVE and 15 mL of HVE were incorporated, mixing continuously until homogenization in a water bath at 40 °C. To this was added 15 mL of oily extract of propolis, 15 mL of hydro-alcoholic extract of propolis, 2 g of apilarnil, and 50 g of honey. The oily and hydro-alcoholic propolis extracts were prepared according to the described method [13].

#### 4.3.2. Preparation of the Ointment with Vegetable Extracts and Polymers

A total of 15 mL OVE and 15 mL HVE were gradually added to 100 g ointment base and mixed until complete homogenization in a water bath at 40 °C. Later, 0.5 g collagen and 0.24 g chitosan were added (previously solubilized by the described method), along with 3 g of lyophilized egg white [13].

#### 4.3.3. Preparation of the Ointment with Bee Products and Polymers

A total of 15 mL of oily extract of propolis, 15 mL of hydro-alcoholic extract of propolis, 2 g of apilarnil, and 50 g of honey were gradually added to 100 g of the ointment base, stirring continuously until homogenized in a water bath at 40 °C. After this stage, 0.5 g of collagen and 0.24 g of chitosan (solubilized in advance) were incorporated, and 3 g of lyophilized egg white were used [13,14].

#### 4.3.4. Preparation of the Ointment with Bee Products, Vegetable Extracts, and Polymers

A total of 15 mL of OVE and 15 mL of HVE were gradually included in 100 g of ointment base (50 g of lanolin and 50 g of vaseline) in a water bath at 40 °C., until complete homogenization occurred. Then, 15 mL of oily extract of propolis, 15 mL of hydro-alcoholic extract of propolis, 2 g of apilarnil, and 50 g of honey were added. Finally, 0.5 g of solubilized collagen, 0.24 g of solubilized chitosan, and 3 g of lyophilized egg white were also added, mixing continuously until homogenization.

### 4.4. Chemical Reagents, Bee Products, and Polymers

The ethyl alcohol, chitosan, and collagen were purchased from Sigma-Aldrich (Steinheim, Germany), and the lanolin and vaseline were from Farma Chim (Ploiesti, Romania). The fresh chicken eggs (*Gallus gallus domesticus*) and olive oil were obtained from a commercial source

The honey, apilarnil, and propolis samples were collected from the same apiary, S.C. Stupina SRL (Balanesti, Gorj County, Romania). The samples were taken in different months, as follows: honey in August, apilarnil in April, propolis in April–September, every 25th of the month, making a mixture of the samples taken.

The organoleptic analysis of honey (appearance, consistency, color, taste, and odor) and the physicochemical determinations of honey and apilarnil (total protein, total fats, ash and water content) have been previously reported [14].

### 4.5. Experimental Animals and Study Design

Wistar adult male rats (220–250 g) were divided into six groups, each with 7 rats, and kept in a temperature-controlled room with dark–light cycles. Prior to the creation of the wound, the animals were anesthetized with ketamine (100 mg/kg, ip). Three models of cutaneous lesions (linear incision, circular excision, thermal burn) were performed according to the models documented in the literature. In the incision model, two linear wounds 1 cm long were created using a sterile scalpel on the right and left paravertebral regions of anesthetized animals, through the full thickness of the skin, at equal distance from the spinal column. In the excision wound model, a full-depth skin wound was created by surgically removing a circular portion of skin from the dorsal interscapular area, using an 8 mm biopsy punch. For the thermal burn wound model, a heated metal plate (9 mm × 8 mm, 100 °C) was placed under its own weight onto the skin of anesthetized animals for 9 s, creating a deep partial thickness burn. The injuries were promptly rinsed with normal saline [12,40,41].

Skin lesions of the NC group (negative control) were not treated, while in the OB group (ointment base), rats were treated with the ointment base. The other four groups were treated once a day for 21 days as follows: the AP group was treated with the ointment based on vegetal extracts and bee products; the PPo group was treated with the ointment based on vegetal extracts and polymers; the Apo group was treated with the ointment based on bee products and polymers; the APPo group was treated with the ointment based on mixture of bee products, polymers, and vegetal extracts.

#### 4.5.1. Assessment of Degree of Wound Healing

Macroscopic and clinical evaluation was performed throughout the study, recording the wound area, calculating the wound contraction of the lesion (days 6 and 9) and performing the histopathological examination on days 1, 2, 3, 9, and 21 of the treatment.

The circular lesion area (wound excision) was calculated with the formula A = πr^2^.

The calculation of the elliptical area resulting from the process of contraction of the lesion was calculated according to the formula πa × b/4. In this context, a represents the major axis, while b signifies the minor axis.

The wound contraction rate (WCR) was calculated as a percentage of the original wound size (A0 = 50.27 mm^2^) for each animal according to the formula WCR = (A0 − At)/A0 × 100, where At = the wound size on days 6 and 9.

#### 4.5.2. Histopathological Analysis

The samples collected were processed according to the method described previously [12].

The scoring of the inflammatory infiltrate (S) was based on an adaptation of scores from the literature [42]. To establish the score, 5 microscopic fields were examined with a magnification of 400×, at the level of the papillary and reticular dermis, with the hypodermis and the striated muscle tissue, respectively, chosen as representative for each individual case. The value of the final score was represented by the average value for the 5 fields analyzed. A score of S0 represented no inflammatory infiltrate, a score of S1 represented mild/rare/occasional inflammatory infiltrate (<10 lymphocytes/HPF), a score of S2 represented moderate/focal inflammatory infiltrate (11–30 lymphocytes/HPF), and a score of S3 represented severe inflammatory infiltrate (numerous lymphocytes > 30/HPF).

The thermal burn depth assessment (D) was based on a score adapted from the specialized literature [43,44]. The scoring parameters are as follows: D0 (normal skin), D1 (epithelial necrosis within the epidermis, but basement membrane remains intact), D2 (epithelial necrosis extends beyond the basement membrane, but hair bulbs remain intact), D3 (necrosis of skin appendages and dermal connective tissue), and D4 (extensive necrosis in the hypodermic tissue).

Grading of the infection (G) was based on a score adapted from the specialized literature, as follows: Grade 0—no microorganisms were identified in the sections; Grade I—microorganisms are identified on the surface of the lesion; Grade II—microorganisms affected the superficial dermis; Grade III—microorganisms affected the entire thickness of the dermis; Grade IV—the microorganisms extensively affected the adjacent viable tissues and the hypodermis [45,46].

### 4.6. Statistical Analysis

The data obtained from the wound excision model were analyzed by one-way ANOVA followed by the Bonferroni post-test. The analysis of the statistics was performed using SPSS version 15.0, created by SPSS Inc. based in Chicago, Illinois, USA, with *p* < 0.05 regarded as statistically significant.

## 5. Conclusions

The idea of making an ointment that includes vegetal extracts, bee products, and natural polymers studied in vivo on experimental models showed remarkable results. The APPo group had already achieved very good results in terms of wound contraction by the sixth day of treatment. For all the tested ointments which contained compounds with therapeutic effects, on the ninth day of treatment, the wound contraction was complete. On the ninth day for the incision lesion and on the twelfth day for excision and thermal burn, the re-epithelialization process was finished without scars for all types of injuries, and at day 21, dermal collagenization and restoration of the histological structure occurred for all treated groups.

## Figures and Tables

**Table 1:** Evaluation of wound area, re-epithelialization area, and wound contraction rate (WCR) for the excision model.

Experimental Groups	Wound Area (mm^2^)			WCR (%) (Mean ± SEM)	
	Day 0	Day 6	Day 9	Day 6	Day 9
NC Group	64.0 (8.0 × 8.0)	61.6 (7.7 × 8.0)	56.25 (7.5 × 7.5)	5.78 ± 1.86	14.85 ± 2.95
OB Group	64.0 (8.0 × 8.0)	45.5 (6.5 × 7.0)	40.8 (6.0 × 6.8)	26.74 ± 2.13 *	33.79 ± 2.21 *
AP Group	64.0 (8.0 × 8.0)	7.5 (2.5 × 3.0)	1.56 (1.2 × 1.3)	89.33 ± 0.60	97.82 ± 0.13
PPo Group	64.0 (8.0 × 8.0)	19.5 (3.0 × 3.5)	2 (1.0 × 2.0)	84.06 ± 0.47	96.61 ± 0.30
APo Group	64.0 (8.0 × 8.0)	9.0 (3.0 × 3.0)	1.5 (1.0 × 1.5)	86.25 ± 0.31	97.50 ± 0.09
APPo Group	64.0 (8.0 × 8.0)	5.0 (2.0 × 2.5)	1.0 (1.0 × 1.0)	91.35 ± 0.83	98.17 ± 0.15

* *p* < 0.001 (the *p* value results as a comparison between row 2 (OB group) and rows 3–6 (AP Group, PPo Group, APo Group, and APPo Group). NC group (negative control group—not treated), OB group—ointment base group (treated with the ointment base), AP group (treated with bee products and vegetal extracts ointment), PPo group (treated with vegetal extracts and natural polymers ointment), APo group (treated with bee products and natural polymers ointment), APPo group (treated with the ointment based on a mixture of bee products, vegetal extracts, and natural polymers).

**Table 2:** Microscopic histology images of the tissue samples on day 1.

Incision	Excision	Thermal Burn
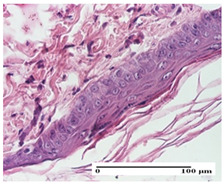	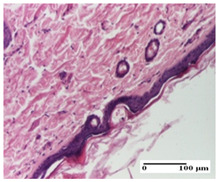	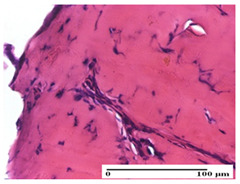
mild inflammatory infiltrate in superficial dermis (S1, G0)	no inflammation, moderate edema (S0, G0)	dermal collagenization, thinning of the epidermis (S0, G0, D1)

Inflammatory infiltration scoring: S0 (no inflammatory infiltrate), S1 (mild inflammatory infiltrate). Thermal burn depths (D): D1—epithelial necrosis within the epidermis, the basement membrane remains intact. Gradation of infection (G): G0—no microorganisms were identified in the sections.

**Table 3:** Microscopic histology images of the tissue samples on day 2.

Experimental Groups	Incision	Excision	Thermal Burn
NC Group	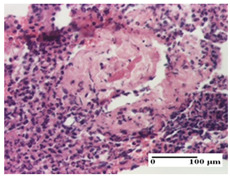	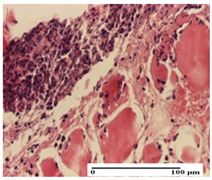	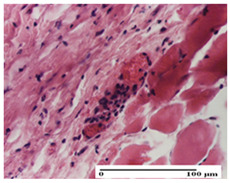
	important inflammatory infiltrate in hypodermis and striated muscle (S3, G0)	deep ulceration and polymorphic inflammatory infiltrate near the striated muscle (S3, G0)	important inflammatory infiltrate in hypodermis and striated muscle (S2, G0, D1)
OB Group	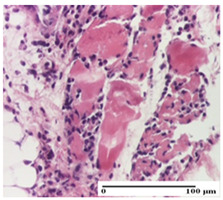	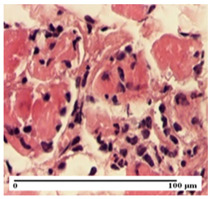	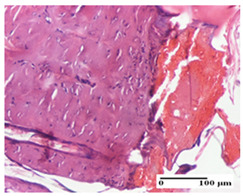
	relatively important inflammatory infiltrate in hypodermis and striated muscle (S2, G0)	moderate polymorphic inflammatory infiltrate in the interstitium of the striated muscle (S2, G0)	dermal collagenization, hemorrhage (S1, G0, D2)
AP Group	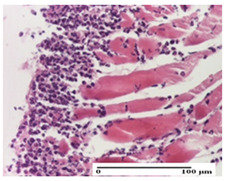	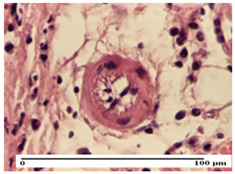	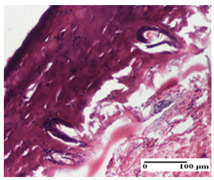
	inflammatory infiltrate in the interstitium of the striated muscle (S3, G0)	thick-walled vessel (S1, G0)	dermal collagenization (S1, G0, D2)
PPo Group	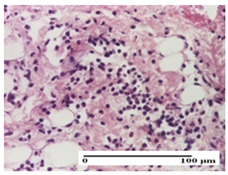	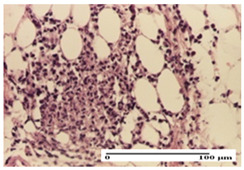	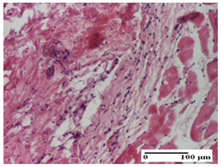
	severe polymorphic inflammatory infiltrate in hypodermis (S2, G0)	polymorphic inflammatory infiltrate in hypodermis (S2, G0)	slight inflammatory infiltrate (S1, G0, D0)
APo Group	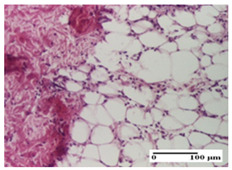	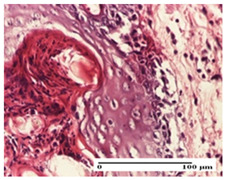	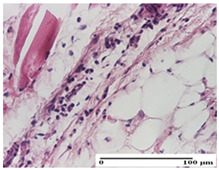
	mild inflammatory infiltrate in hypodermis (S1, G0)	abscess in the corneous layer, epidermal acanthosis, moderate polymorphic inflammatory infiltrate in dermis (S2, G0)	mild inflammatory infiltrate in hypodermis and between the striated muscle fibers (S1, G0)
APPo Group	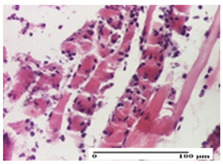	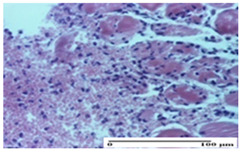	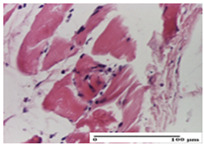
	moderate interstitial inflammatory infiltrate (S1, G0)	moderate inflammatory infiltrate in the interstitium of the striated muscle (S1, G0)	inflammatory infiltrate in the striated muscle (S1, G0, D0)

Inflammatory infiltration scoring: S0 (no inflammatory infiltrate), S1 (mild inflammatory infiltrate), S2 (moderate inflammatory infiltrate), S3 (severe inflammatory infiltrate). Thermal burn depths (D): D0—normal skin, D1—epithelial necrosis within the epidermis, the basement membrane remains intact; D2—epithelial necrosis extends beyond the basement membrane, but hair bulbs remain intact. Gradation of infection (G): G0—no microorganisms were identified in the sections.

**Table 4:** Microscopic histology images of the tissue samples on day 3.

Experimental Groups	Incision	Excision	Thermal Burn
NC Group	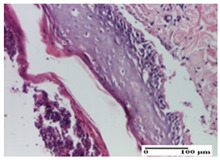	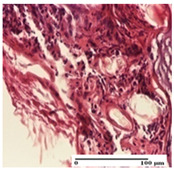	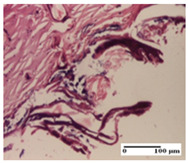
	abscess in the corneous layer, epidermal acanthosis, mild inflammatory infiltrate in dermis (S2, G0)	superficial ulceration (S3, G0)	abscess in the corneous layer, mild dermal inflammatory infiltrate (S1, G0, D2)
OB Group	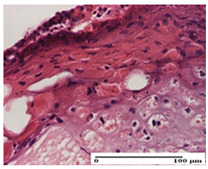	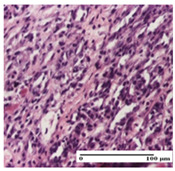	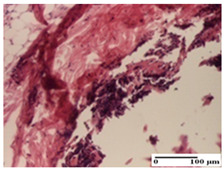
	degeneration of keratinocytes, paranucleosis, vacuolar spongiosis (S2, G0)	inflammatory infiltrate in hypodermis (S3, G0)	abscess in the corneous layer (S3, G0, D2)
AP Group	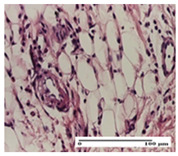	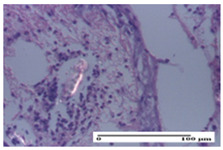	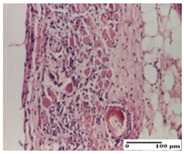
	hypodermis with mild inflammatory infiltrate (S1, G0)	lymphocytic inflammatory infiltrate—detail in polarized light microscopy (S2, G0)	fibrosis that embeds striated muscle fibers, mild inflammation (S1, G0, D2)
PPo Group	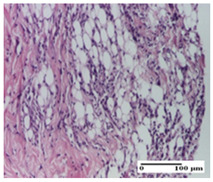	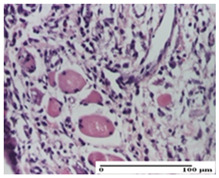	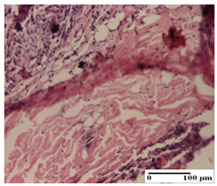
	moderate inflammatory infiltrate in the dermis and hypodermis (S2, G0)	moderate inflammatory infiltrate in hypodermis and striated muscle (S2, G0)	abscess in the corneous layer (S2, G0, D2)
APo Group	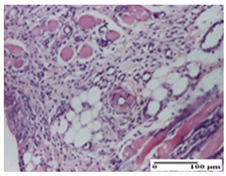	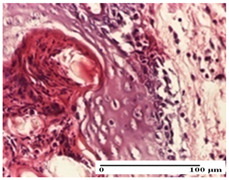	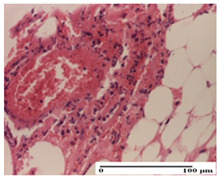
	moderate inflammatory infiltrate (S2, G0)	abscess in the corneous layer moderate vacuolization (S2, G0)	congestion and hemorrhage (S1, G0, D0)
APPo Group	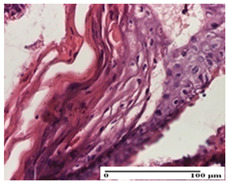	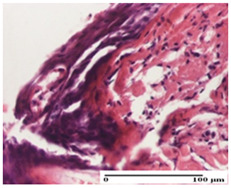	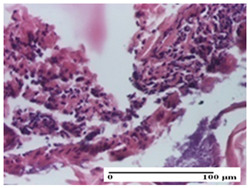
	severe inflammatory infiltrate in the dermis, micro-ulceration of the epidermis with hematoleukocyte surface deposit (S3, G0)	inflammatory infiltrate (S1, G0)	Inflammatory infiltrate (S3, G0, D2)

Inflammatory infiltration scoring: S0 (no inflammatory infiltrate), S1 (mild inflammatory infiltrate), S2 (moderate inflammatory infiltrate), S3 (severe inflammatory infiltrate). Thermal burn depths (D): D0—normal skin, D2—epithelial necrosis extends beyond the basement membrane, but hair bulbs remain intact. Gradation of infection (G): G0—no microorganisms were identified in the sections.

**Table 5:** Microscopic histology images of the tissue samples on day 9.

Experimental Groups	Incision	Excision	Thermal Burn
NC Group	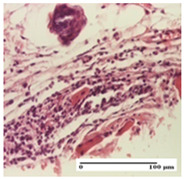	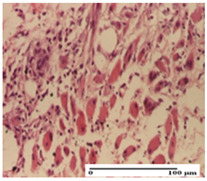	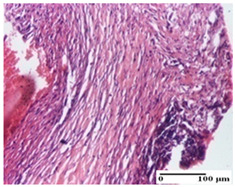
	important inflammatory infiltrate in hypodermis (S3, G0)	edema (striated muscle) (S1, G0)	abscess, ulceration (S2, G0, D2)
OB Group	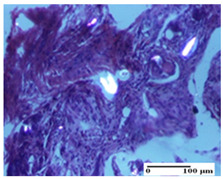	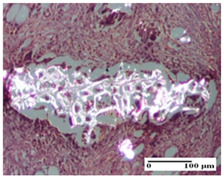	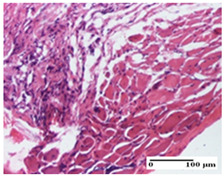
	foreign body-type giant cell response to keratin (S1, G0)	foreign body-type giant cell response to keratin—detail in polarized light microscopy (S1, G0)	mild inflammatory infiltrate (S1, G0, D0)
AP Group	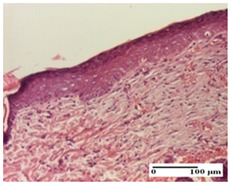	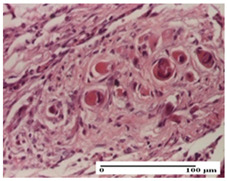	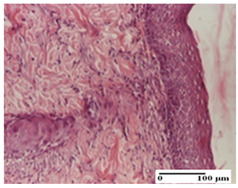
	dermal collagenization (S0, G0)	severe dermal collagenization that included the muscle layer (S1, G1)	severe dermal collagenization (S1, G0, D0)
PPo Group	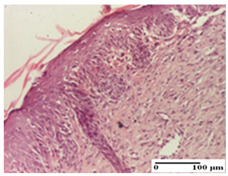	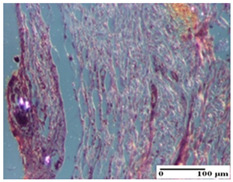	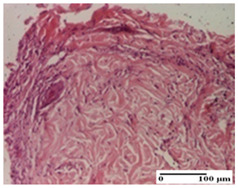
	dermal collagenization (S0, G0)	dermal collagenization—detail in polarized light microscopy (S0, G0)	dermal collagenization (S0,G0, D0)
APo Group	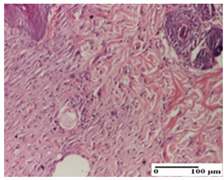	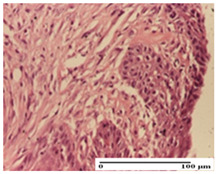	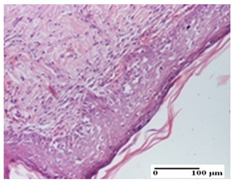
	dermal collagenization (S0, G0)	dermal collagenization, epidermal acanthosis (S0, G0)	moderate dermal collagenization, mild inflammatory infiltrate (S1, G0, D0)
APPo Group	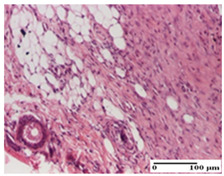	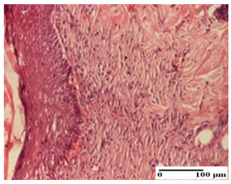	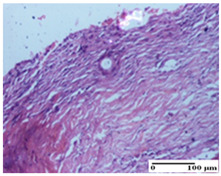
	Dermo-hypodermic collagenization, mild inflammatory infiltrate (S1, G0)	dermal collagenization (S0, G0)	dermal collagenization, rare inflammatory elements—detail in polarized light microscopy (S1, G0, D0)

Inflammatory infiltration scoring: S0 (no inflammatory infiltrate), S1 (mild inflammatory infiltrate), S2 (moderate inflammatory infiltrate), S3 (severe inflammatory infiltrate). Thermal burn depths (D): D0—normal skin. Gradation of infection (G): G0—no microorganisms were identified in the sections, G1—microorganisms are identified on the surface of the lesion.

**Table 6:** Microscopic histology images of the tissue samples on day 21.

Experimental Groups	Incision	Excision	Thermal Burn
NC Group	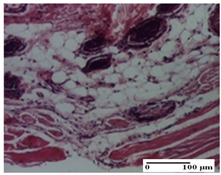	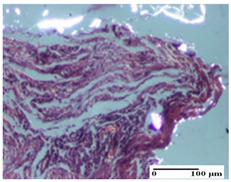	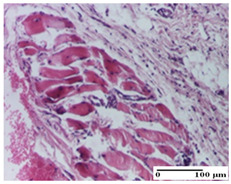
	inflammatory infiltrate in hypodermis and muscle tissue (S1, G0)	ulceration, important inflammatory infiltrate (S2, G0)— detail in polarized light microscopy	striated muscle with interstitial inflammatory infiltrate (S1, G0, D0)
OB Group	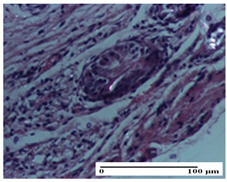	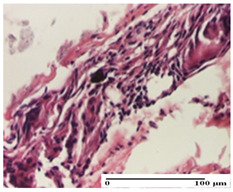	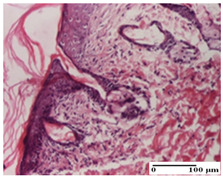
	inflammatory infiltrate and fibrosis in hypodermis, foreign body-type giant cell response to keratin (S2, G0)	relatively important inflammatory infiltrate (S2, G0)	congestion and edema, mild dermal collagenization (S1, G0, D0)
AP Group	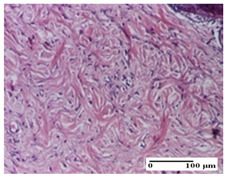	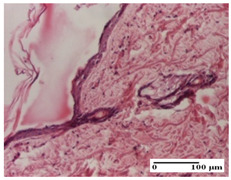	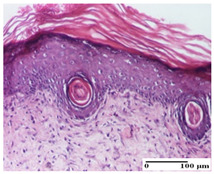
	important collagenization of the dermis (S1, G0)	dermal collagenization, mild edema of the dermis (S1, G0)	severe collagenization of the dermis (S1, G0, D0)
PPo Group	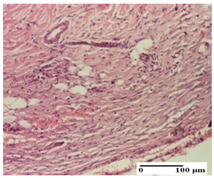	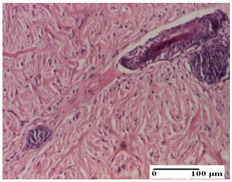	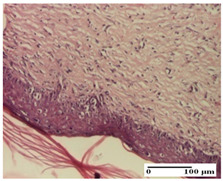
	dermal collagenization (S1, G0)	dermal collagenization (S1, G0)	dermal collagenization (S1, G0, D0)
APo Group	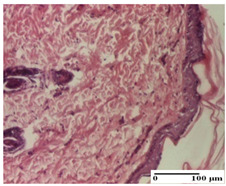	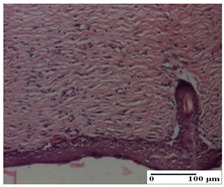	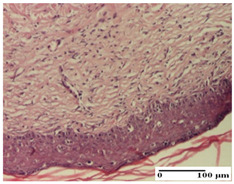
	dermal collagenization (S0, G0)	dermal collagenization, the hair follicle with atrophy of the sebaceous glands (S1, G0)	dermal collagenization (S1, G0, D0)
APPo Group	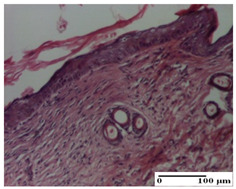	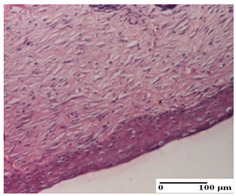	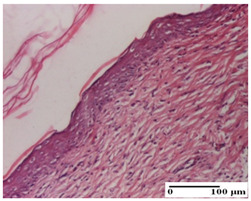
	dermal collagenization, atrophy of skin appendages (S1, G0)	dermal collagenization (S0, G0)	dermal collagenization (S1, G0, D0)

Inflammatory infiltration scoring: S0 (no inflammatory infiltrate), S1 (mild inflammatory infiltrate), S2 (moderate inflammatory infiltrate). Thermal burn depths (D): D0—normal skin. Gradation of infection (G): G0—no microorganisms were identified in the sections.

## Data Availability

The data presented in this study are available on request from the corresponding authors.

## Supplementary Materials

The following supporting information can be downloaded at: https://www.mdpi.com/article/10.3390/ph18010065/s1, Table S1. Macroscopic pictures of the cutaneous lesions.
