# Supplementary material for: In Vivo Assessment of Healing Potential of Ointments Containing Bee Products, Vegetal Extracts, and Polymers on Skin Lesions
Source: Pharmaceuticals (Basel). 2025 Jan 9;18(1):65. doi: 10.3390/ph18010065 (PMC11768340; doi:10.3390/ph18010065)
Supplement: Supplementary file 1 [file pharmaceuticals-18-00065-s001.zip › pharmaceuticals-3391963-supplementary.pdf]

Table S1. Macroscopic pictures of the cutaneous lesions

	AP Group			APo Group		
	Incision	Excision	Thermal burn	Incision	Excision	Thermal burn
Day 0	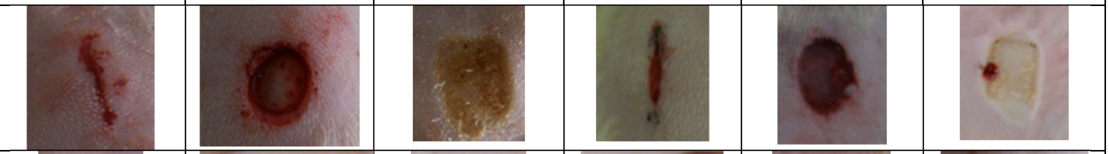					
Day 1	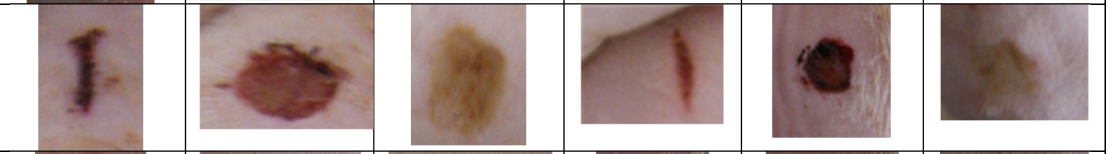					
Day 2	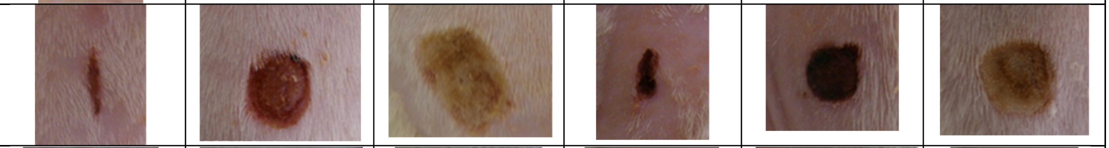					
Day 3	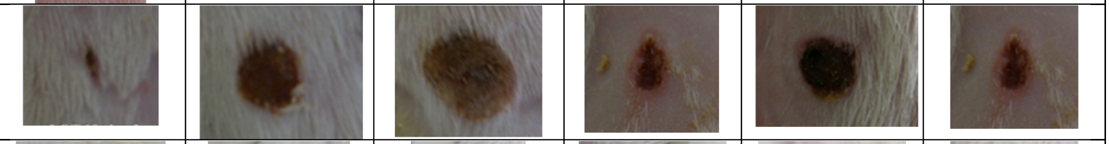					
Day 6	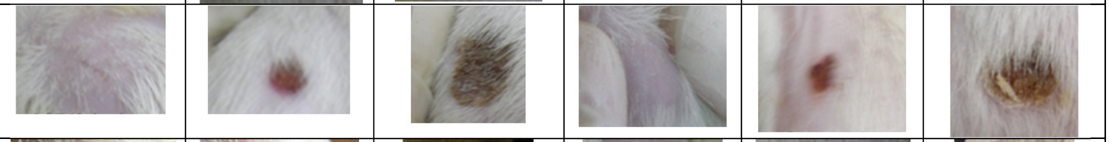					
Day 9	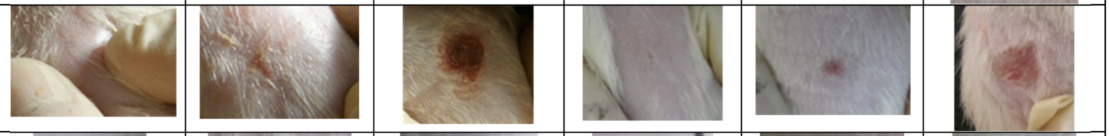					
Day 12	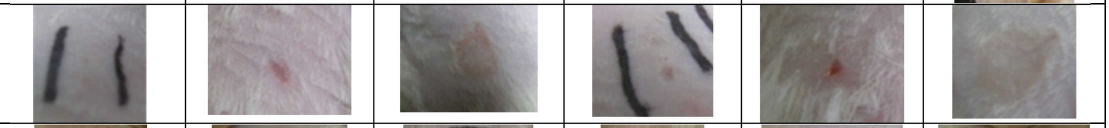					
Day 21	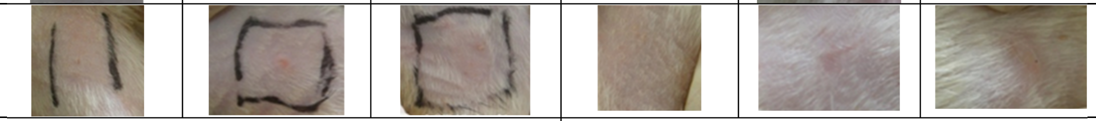					
	PPo Group			APPo Group		
Day 0	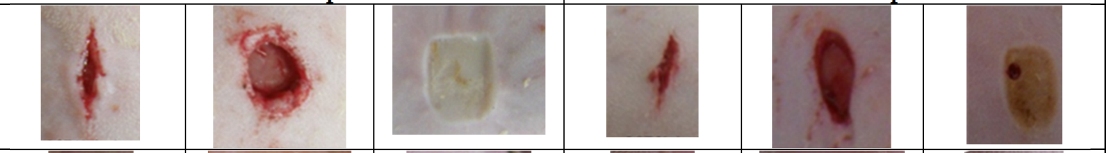					
Day 1	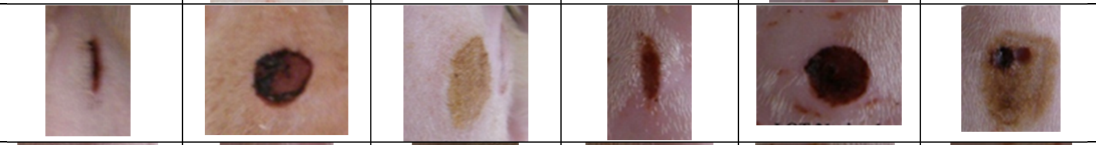					
Day 2	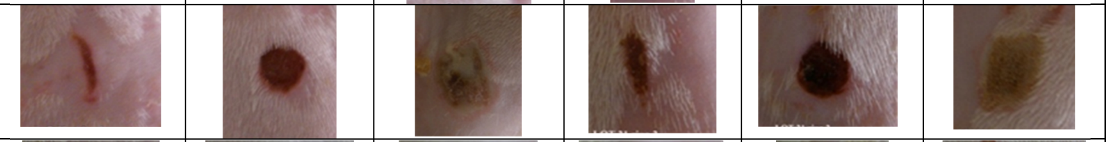					
Day 3	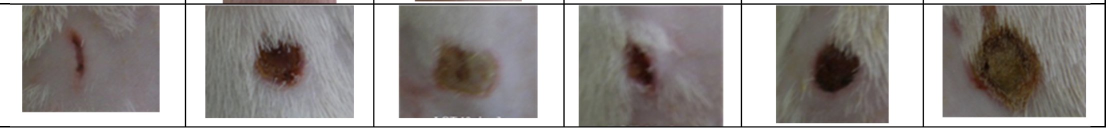					

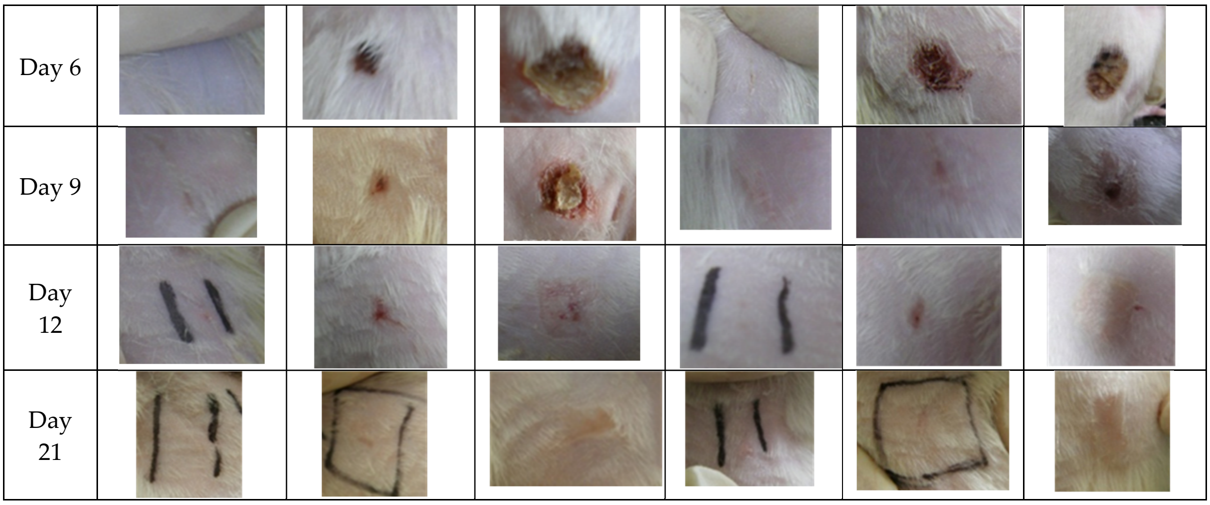

AP group (treated with bee products and vegetal extracts ointment), PPo group (treated with vegetal extracts and natural polymers ointment), APo group (treated with bee products and natural polymers ointment), APPo group (treated with the ointment based on mixture of bee products, vegetal extracts and natural polymers).
